# Consumption Rate of Lichens by *Constrictotermes cyphergaster* (Isoptera): Effects of C, N, and P Contents and Ratios

**DOI:** 10.3390/insects10010023

**Published:** 2019-01-09

**Authors:** Ana M. Barbosa-Silva, Alexandre Vasconcellos

**Affiliations:** Laboratório de Termitologia, Departamento de Sistemática e Ecologia, Universidade Federal da Paraíba, João Pessoa 58051-900, Paraíba, Brazil; avasconcellos@dse.ufpb.br

**Keywords:** stoichiometry, decomposition, isoptera, neotropical region, semi-arid

## Abstract

Wood is the main dietary item for most termites; however, supplementation with certain nutrients may occur via the ingestion of other available food resources in the ecosystem. The objective of this study was to evaluate the consumption of lichens with different C, N, and P contents by *Constrictotermes cyphergaster* under laboratory conditions, and estimate the intake of this substrate by this species in a semi-arid area of Northeast Brazil. The foraging activities of fifteen field colonies were monitored over 15 days from 6:00 p.m. to 6:00 a.m., and the lichens that were consumed were identified. Blocks of lichen thallus (1.5 × 1.5 cm) of four lichen species were offered to the termites in the laboratory. The mean total consumption rate of lichen by *C. cyphergaster* was 0.032 mg lichen/g termite (fresh weight)/day. *Dirinaria confluens* was the lichen most consumed by termites (0.010 mg lichen/g of termite (fresh weight)/day), followed by *Lecanora* spp. and *Haematomma persoonii* at a mean consumption of 0.008 and 0.006 mg lichen/g termite (fresh weight)/day, respectively. Based on the size of the *C. cyphergaster* populations, the estimated lichen consumption rate was 105.12 g lichen/ha/year. Lichen consumption was significantly affected by the N content and the C:N and C:P ratios, with the N content being the factor that best explained the consumption by the termites. The results suggest that *C. cyphergaster* can use lichens as a supplemental source of nutrients, especially nutrients that are found in low concentrations in wood.

## 1. Introduction

Termites are macroarthropods that act as important mediators of organic matter decomposition and energy and nutrient fluxes in terrestrial ecosystems [1,2]. These insects can consume 14 to 50% of the annual plant necromass production, with values reaching 100% in some deserts [2,3].Termite consumption in the field is difficult to estimate, mainly because of the cryptic foraging behavior of most termite species [4,5]. Studies under laboratory conditions have been conducted and improved for decades [6,7,8,9], and they have been a widely used strategy for understanding various environmental parameters, including food choice and consumption rates [10,11,12,13,14,15,16].

Termites consume a variety of organic material including wood, grass, herbs, litter fungi, lichens, leaves, roots, animal excrement, and even decaying animal carcasses, although a cellulosic diet is most common [4,17]. The consumption of wood by termites is characterized by a food preference gradient ranging from live wood to wood in advanced stages of decomposition [18,19]. The plant substrates are rich in carbon and hydrogen, but they are considered nutritionally poor, especially in nitrogen and phosphorus [20,21,22], which are essential for the production of proteins, ATP, and mRNA [23]. The C:N ratio found in wood is 100 times higher than that found in the body of termites [24]. The concentration of N in termite tissues is estimated at 8 to 13%, whereas in wood, this content is 0.5% [25,26]. To adjust the relationship between nutrients, termites have developed resource differentiation mechanisms, and they preferentially feed on substrates with higher nitrogen contents [27,28]. A common strategy among these insects is the preference for decomposing plant material, due to the presence of microorganisms (mainly fungi) that can provide additional sources of nitrogen [29] and vitamins [30]. Food preference tests showed that N and P are phagostimulants in the termite diet [11,31]. Properties such as hardness (density) and secondary compound contents can also be determinants of the choice and consumption of food items [32,33,34,35].

Similar to termites, ants select N-rich resources, mainly for feeding the larvae and the queen. Omnivorous ants may select their food in response to nutrient imbalances, and forage in a way that optimizes complementary nutrition. The CHO:N protein balance seems to be vital to these insects, and it has been the subject of previous studies [36,37,38]. The balance of P:C has been recognized as a mediator of ant social interactions, with diets rich in carbohydrates (low P:C) increasing the aggressive activity of workers in colonies [39].

Species of *Hospitalitermes*, *Grallatotermes*, *Longipeditermes* and *Constrictotermes* have added lichens to their diet, which are suggested to represent an extra source of N for termites, and they are also good retainers of phosphorus and other nutrients [28,40,41,42,43,44,45]. According to Collins [40], *Hospitalitermes umbrinus* can consume up to 50 kg of lichens and mosses/ha/year. In the Neotropical region, the termite *Constrictotermes cyphergaster* (Termitidae, Nasutitermitinae) is distributed in Argentina, Bolivia and Paraguay, and the semi-arid region of Brazil, where it is notable for the abundance of nests (59 active nests/ha) and the consumption of plant necromass [46,47]. The food items consumed by this termite at night on open trails include 29 species of crustose lichens [44,48]. However, the effect of the nutritional composition of lichens on the termite diet remains unknown. Understanding this trophic interaction between lichens and termites from a qualitative and quantitative point of view may provide insights into the pathways of some nutrients, and the functional role of termites in the ecosystem.

The objectives of this study were to evaluate the consumption and preference of *C. cyphergaster* for lichens with different concentrations of N, C, and P, and different C:N, C:N:P, N:P, and C:P ratios. These three elements are important to many biological processes, but they are relatively rare in the environment. The relative balance of elements that are available in food can influence how species live and reproduce within a particular environment [23]. Furthermore, the balance of these elements may be equally or perhaps even more important than the absolute amount of any one nutrient [23,49]. Lichen consumption by *C. cyphergaster* was estimated in an area with Caatinga vegetation, a type of seasonally dry tropical forest (SDTF) of South America that is found in the semi-arid domain of Northeast Brazil. 

## 2. Materials and Methods

### 2.1. Study Area

This study was conducted at Fazenda Almas Natural Heritage Private Reserve (RPPN Fazenda Almas) (7°28′15″S 36°53′51″W), which encompasses an area of approximately 3505 ha. The mean annual rainfall in the region is 560 ± 230 mm, which is concentrated in the months of February, March, and April, and a long dry season, which often lasts more than eight months, also occurs. The annual mean temperature and relative humidity are 25°C and 65%, respectively [50,51].

### 2.2. Collection and Sampling Procedures

**Figure 1:**
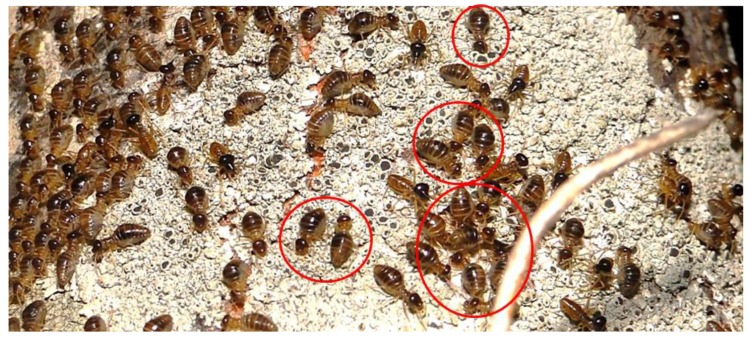
Consumption of *Dirinaria confluens* by *Constrictotermes cyphergaster* at RPPN Fazenda Almas, Paraíba, Brazil. Circles indicate workers displaying characteristic feeding behavior.

Fifteen field nests of *C. Cyphergaster*, were randomly selected for observation of lichen foraging. The nests were separated by a minimum distance of 20 m, and they were monitored for 15 consecutive days from June 5 to June 20, 2017, with observations performed at each hour from 6:00 p.m. to 6:00 a.m. [48]. At each observation, the occurrence of lichen consumption by termites was noted. Lichen consumption was considered to be positive when workers were recorded, forming groups (minimum of three individuals) on lichen thalli, with a curved head indicative of contact with the food and abdominal constriction movements, indicative of individuals performing collection (Figure 1). The exploited lichen thalli were tagged with ethyl vinyl acetate (EVA) labels, and at the end of the experiment, they were collected for taxonomic identification. Each lichen was marked only once. The study did not consider the termite trails formed in the canopy layer because of the difficulty of observation; thus, the observations were limited to the lower portion of the vegetation (up to 2 m high). 

### 2.3. Lichen Species Determination

The species were identified by consulting Cáceres, Lücking, and Rivas-Plata and Marbarch [52,53,54]. The thallus characteristics, such as habit, ascoma shape and type, color, size, and ascospore septation, were evaluated. Cross-sections of the thallus and ascoma were stained with iodine (I) and/or potassium hydroxide (KOH) to determine the presence of lichen compounds of taxonomic importance [55].

### 2.4. Macronutrient Quantification

The C, N, and P content in the thalli of *Dirinaria confluens* (Fr.) D. D. Awasthi, *Pertusaria flavens* Nyl., *Lecanora* spp., and *Haematomma persoonii* (Fée) A. Massal were determined. Approximately 25 stalks of each lichen species were used in the analysis. These species were selected because they contain a greater amount of biomass in their thalli compared to the other lichens. Thalli with low biomass could not be analyzed for nutrient contents, because the lichen could not be separated from the substrate. The collected lichens were individually scraped from the substrate with a steel blade, and the obtained biomass (2 g) was packed in glass containers. 

The method of Walkley & Black [56] was used to determine C, and that of Kjeldahl [57] for N and P, both with the modifications of Tedesco et al. [58]. A total of 100 mg of lichen biomass was used for quantifying C. Oxidation of organic matter was performed by using potassium dichromate in the presence of sulfuric acid, with heat being provided by the sulfuric acid, and from external heating (boiling for 5 min). A total of 200 mg of the sample was used for the quantification of N and P, which was digested in the presence of hydrogen peroxide and sulfuric acid (H_2_SO_4_). All nutrient analyses were performed in triplicate.

### 2.5. Lichen Consumption in the Laboratory

Termites from five nests were collected for consumption bioassays, and three subcolonies were separated from each nest. Each bioassay was composed of 200 workers and 50 soldiers [12], which were removed from the nests with the help of popsicle sticks. The individuals were placed in two cylindrical glass containers (6 cm in diameter at the base and 8 cm in height, volume ~200 mL) that were connected by glass tubes (10 cm) made from serological pipettes. The termites were placed in one of the containers, which was lined with 1 cm of sifted, washed, and sterilized sand covered with 0.5 cm of expanded vermiculite, moistened with 8 mL of sterilized distilled water (adapted from [59]). The other glass container was lined with 1 cm of sand and lichen thallus fragments (1.5 × 1.5 cm), with one of each species (*D. confluens*, *P. flavens*, *Lecanora* spp., and *H. persoonii*) placed at the cardinal points. The basal part of the lichens was wrapped with aluminum foil to prevent the termites from eating wood remnants from the plant supporting the lichen. The glass tube that connected the containers ended at the center of the container with the lichens, to ensure that the distance between each lichen and the center of the arena was equivalent. The containers were covered with voile fabric secured with a rubber band, and placed in total darkness for 14 days at room temperature. The bioassay time was determined from previously performed survival tests that indicated that after 14 days, significant increases in termite mortality rates occurred.

Every three days, the bioassays were observed, and the dead individuals were carefully removed and counted. Three treatments that had mortality greater than 25% at the end of the experiment were discarded. A 2 mL aliquot of sterile distilled water was added to the subcolonies with each cleaning procedure. Five containers with lichen fragments but without termites were used as controls.

### 2.6. Data Analysis

Lichen consumption was calculated, as the difference between the initial and final weight of the thalli, which was corrected, whenever necessary, by the weight loss of the controls (containers without termites) [60,61]. The consumption values obtained in each arena were transformed to mg of consumed lichen/g fresh termites/day. The participation of *C. cyphergaster* in total lichen consumption per hectare was estimated from data obtained by Vasconcellos et al. [46], who recorded the population density and biomass of *C. cyphergaster* in the area as 272 individuals/m^2^ and 0.9 g (fresh weight)/m^2^, respectively. The differences between lichen consumption, and the content and ratio of macronutrients (C, N, P) were evaluated by analysis of variance (ANOVA) and an a posteriori Bonferroni’s test. Two independent models were constructed to test the effects of lichen nutrient composition on consumption by *C. cyphergaster*: one for the nutrients (C, N, P) and another for their relative ratios (C:N, C:N:P, C:P, and N:P). Deviation analysis (ANODEV) was used to generate generalized linear models (GLMs). The data were analyzed with a Gaussian error distribution with identity link. Residual tests were performed to determine the error distribution and model fit. The adjusted pseudo-R^2^ values were calculated using the vegan package. All analyses were performed using the statistical program R version 3.2 [62].

## 3. Results

### 3.1. Lichens Consumed by *C. cyphergaster* in the Field

Fourteen species of crustose lichens composed the diet of *C. cyphergaster* in the field: *Arthothelium* sp., *Arthonia* sp., *Anisomeridium tamarindii* (Fée) RC Harris, *Anisomeridium* sp. *Chrysothrix xanthina* (Vain.) Kalb, *Dirinaria confluens*, *Glyphis scyphulifera* (Ach.) Staiger, *Haematomma persoonii*, *Lecanora* sp., *Lecanora achroa* Nyl., *Lecanora leprosa* Fée, *Lecanora tropica* Zahlbr, *Pertusaria flavens*, and *Physcia integrata* (Nyl.).

### 3.2. Quantification of Lichen Consumption in the Laboratory

The mean total lichen consumption under laboratory conditions rate by *C. cyphergaster* was 0.032 mg lichen/termite g (fresh weight)/day (Table 1), representing a consumption rate of 105.12 g of lichen/ha/year. The consumption rates among the lichens offered in the bioassays were significantly different (F_3,8_ = 7.05, *p* < 0.001). *D. confluens* was the lichen species most consumed by the termites (0.010 mg lichen/g of termite (fresh weight)/day), followed by *Lecanora* spp. and *H. persoonii*, with means of 0.008 and 0.006 mg of lichen/g termite (fresh weight)/day, respectively (Table 1).

### 3.3. Concentration of Nutrients in Lichens and Their Effect on Consumption

**Figure 2:**
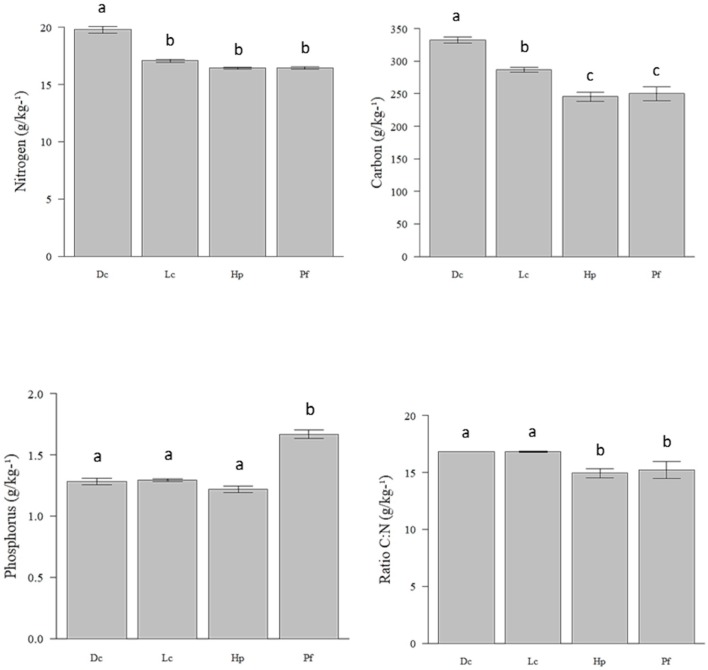

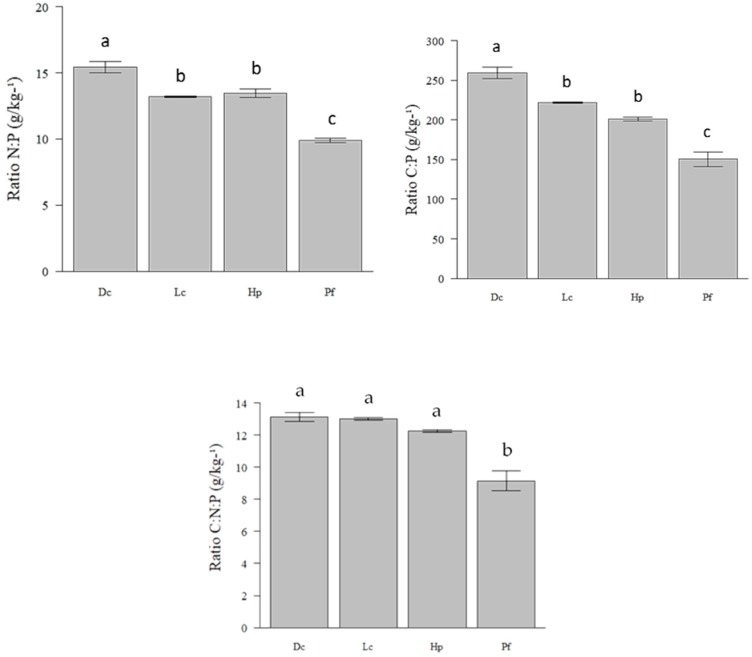
C, N, and P contents and C:N, C:N:P, N:P, C:P ratios (g/kg) of *Dirinaria confluens* (Dc), *Lecanora* spp. (Lc), *Haematomma persoonii* (Hp), and *Pertusaria flavens* (Pf) consumed by *Constrictotermes cyphergaster* in a semi-arid area in Northeast Brazil. Different letters indicate statistically significant differences according to Bonferroni’s test.

The species *D. confluens*, *Lecanora* spp., *H. Persoonii*, and *P. flavens* differed in their C (F_3,8_ = 33.19; *p* < 0.01), N (F_3,8_ = 76.93; *p* < 0.05) and P (F_3,8_ = 58.96; *p* < 0.05) contents, and C:N (F_3,8_ = 5.65, *p* < 0.05), C:N:P (F_3,8_ = 29.43; *p* < 0.05), N:P (F_3,8_ = 66.26, *p* < 0.05), and C:P (F_3,8_ = 58.02, *p* < 0.05) ratios (Figure 2).

Lichen consumption was significantly affected by the N content (ANODEV, GLM: *p* < 0.001), and C:N (ANODEV, GLM: *p* < 0.001) and C:P (ANODEV, GLM: *p* < 0.01) ratios (Table 2). These nutrients showed higher contents in *D. confluens* and *Lecanora* spp., which are species that are most comonly consumed by *C. Cyphergaster* nos bioensaios (Table 1 and Figure 2). The lichen property that best explained termite consumption was the N content (pseudo-*R*^2^ = 0.32), followed by the C:N (pseudo-*R*^2^ = 0.23) ratio.

## 4. Discussion

The lichen diet of *C. cyphergaster* was composed mainly of crustose lichens, as was observed for *Hospitalitermes umbrinus* in Malaysia [40]. There are no records foliose or fruticose lichens being consumed by termites, suggesting that these insects are adapted to consuming crustose lichens. The species recorded as consumed in the present this study are common in the Caatinga environment, and 11 of them were have been previously recorded on the diet of *C. cyphergaster* in Brazilian semi-arid areas [44,63]. The lichens *Arthothelium* sp., *Haematomma persoonii*, and *Physcia integrata* represent new records for consumption by *C. cyphergaster*.

The estimated lichen consumption by *C. cyphergaster* (105.12 g of lichen/ha/year) was small when compared to the consumption of this resource by *H. umbrinus* (50 kg of lichens and moss ha/year) in Malaysia [40]. Lichens are the main component of the diet of *Hospitalitermes* species [40], whereas this resource is only one of the food items consumed by *C. cyphergaster*. Trunks and branches at different stages of decomposition represent the main food item for *C. cyphergaster* [48], and the estimated consumption is 44.5 kg/ha/year [47]. It should also be pointed out that the studies of consumption by *Hospitalitermes* took place in the field, thus explaining the greater range of lichens consumed.

Our results showed a C:N ratio between 14.4 and 16.9 in the lichens offered to the termites. Higher ratios at between 17 and 29 were found for *Croton* spp. (Baill., Kunth, Lam), *Mimosa* spp. (Harms., Mart. Ex Benth., Barneby, (Willd.) Poir.), and *Poincianella pyramidalis* ((Tul.) L.P. Queiroz) in the diet of *C. cyphergaster* [64] (see Supplementary Material Table S1). Higher N contents in lichens compared to wood were also observed in the diet of *Hospitalitermes* spp., with 10 to 60 times more N recorded in lichen thalli than in wood [28], indicating that lichens are a good alternative for N supplementation in termite diet. Field observations made during this study, showed that the foraging of *C. cyphergaster* is not directed to lichens, and this resource is occasionally collected when it is found to be close to the branches and trunks consumed, or along the route between the nest, and the main resource consumed.

Nitrogen was the element that best explained the consumption of lichens by termites (pseudo-R^2^ = 0.32), although the species *D. confluens* and *Lecanora* spp., which presented higher C:N ratios, were the most highly consumed. According to the C:N equilibrium hypothesis, some termites do not eliminate carbon from their diet, but rather seek a source of nitrogen-rich food to meet their energy needs [21]. A simple approach to dealing with elemental imbalance is to assimilate only a fraction of the nutrient that is abundant in the resource and all of the nutrient that is limiting [24]. In this sense, termites may obey a processing rule in which 1% of the ingested C, and all of the available N are assimilated [24], which would explain the consumption of lichens with high C contents as well, suggesting that the cost-benefit of this interaction is compensated by the supply of N.

For wood-consuming termites, N is a limited resource, and several mechanisms to obtain this nutrient have evolved. These mechanisms include preferential assimilation of atmospheric N through bacteria in their digestive tract, consumption of nitrogen rich-foods, the cultivation of fungus gardens, which is characteristic of Macrotermitinae species, and the consumption of wood infected with these microorganisms [30,65]. The association with N-fixing bacteria is present from the most basal families of termites, such as Mastotermitinae and Kalotermitidae, to the most derived ones, such as Macrotermitinae, Termitinae, and Nasutitermitinae [66]. Cannibalism, necrophagy, and trophallaxis are also common in several taxa, and they are believed to be effective means of preventing N loss [65,67]. Physiological evidence of uric acid retention in termite fat bodies has also been reported as a mechanism for retaining nitrogen stores [68,69].

The P content, and the C:P and C:N:P ratios in the lichens had no effect on consumption by *C. cyphergaster*, and the N:P ratio (pseudo-R^2^ = 0.06) did not have an influence on termite consumption, indicating that P may not have an influence on lichen consumption. Moreover, the P content in lichen thalli, in combination with the C and N content, reach ratios that are unfavorable for consumption. In invertebrates, C:N:P ratios are associated with important ecological processes, such as decomposition and N^2^-fixing ability, whereas the C:P and N:P ratios affect the synthesis of ribosomal RNA and the growth rates of organisms [23,70]. The lack of a relationship between lichen consumption and P content in this substrate is explained by the fact that *C. cyphergaster* satisfies the requirement for this nutrient with another food source. In the Caatinga and Cerrado environments of Brazil, where *C. cyphergaster* occurs, the soils are rich in P [71], and they may represent the main source of input of this nutrient to the colonies. Although *C. cyphergaster* is not humivorous, this termite frequently carries soil particles in its jaws to build its nests [72], and thus, is in direct contact with soil throughout the life of the colony. A similar observation was made for *Reticulitermes flavipes*, which absorbs nutrients from its food, but is also able to obtain nutrients from the soil [73].

Resource selection by termites is influenced by the energy requirements of the populations. The nutritional requirements of a termite colony are determined by the size of the population, and the production of new individuals, which follows a seasonal temporal pattern [74,75]. Ants also exhibit a seasonal response to a variety of nutrients [76,77]. Similarly, termites can adjust their eating behavior as seasons change [78,79]. For example, seasonal responses based on phosphate availability were observed for *R. flavipes* Kollar [11]. Due to these aspects, the quality and quantity of the substrate consumed in the ecosystem under natural conditions may be different from those observed in the laboratory, and consequently, may influence consumption rates.

## 5. Conclusions

A comparison between lichen and wood consumption by *C. cyphergaster* indicates that lichens are a complementary resource in the diet of this termite. However, the choice of the consumed lichen is apparently non-random, with the N content being a factor influencing the consumption rate. Ingestion of lichens reveals that the food items of the *C. cyphergaster* diet can be quite varied, and shows how this species can qualitatively and qualitatively affect the pathways of some essential nutrients, such as C and N, in semi-arid ecosystems.

## Figures and Tables

**Table 1:** Mean consumption rate in the laboratory of four lichen species (mg of lichen/g of termite (fresh weight)/day) by *Constrictotermes cyphergaster.*

Lichen	¹ Mean ± SE	Range
*Dirinaria confluens*	0.0106 ± 0.0009 ^a^	0.005–0.015
*Pertusaria flavens*	0.0064 ± 0.0007 ^b^	0.003–0.012
*Haematomma persoonii*	0.0067 ± 0.0007 ^b^	0.003–0.012
*Lecanora* spp.	0.0081 ± 0.0004 ^ab^	0.005–0.012
Total mean consumption	0.0318 ± 0.0009	0.003–0.015

^1^ Different letters indicate statistically significant differences, according to Bonferroni’s test.

**Table 2:** Effect of nutrient contents (C, N, P—Model one) and their ratios (C:N; C:N:P, C:P, N:P—Model Two) on the consumption of lichens by *C. cyphergaster* in the laboratory.

	df	Deviance	Resid. df	Resid. dev	F	Pseudo *R*^2^	P (>F)
N	1	0.0050547	48	0.010547	22.6322	0.32	<0.001
C	1	0.0001990	47	0.010348	0.8908	0.01	0.35
P	1	0.000746	46	0.010274	0.5661	0.00	0.56
CN	1	0.0036748	48	0.11927	16.4539	0.23	<0.001
C:P	1	0.0009845	47	0.010943	4.4080	0.06	<0.01
N:P	1	0.0006689	46	0.010274	2.9951	0.04	0.09
C:N:P	1	0.0001860	46	0.011741	0.7444	0.00	0.39

## Supplementary Materials

The following are available online at http://www.mdpi.com/2075-4450/10/1/23/s1, Table S1: C, N and P contents (g/kg) found in *Poincianella pyramidalis*, *Mimosa* spp., and *Croton* spp., organisms of the diet of *Constrictotermes cyphergaster* in a semi-arid area in northeast Brazil. Five individuals of each species were analyzed.
