# Supplementary material for: Consumption Rate of Lichens by Constrictotermes cyphergaster (Isoptera): Effects of C, N, and P Contents and Ratios
Source: Insects. 2019 Jan 9;10(1):23. doi: 10.3390/insects10010023 (PMC6358737; doi:10.3390/insects10010023)
Supplement: Supplementary file 1 [file insects-10-00023-s001.pdf]

Table S1. C, N and P contents (g/kg) found in *Poincianella pyramidalis*, *Mimosa* spp., and *Croton* spp., organisms of the diet of *Constrictotermes cyphergaster* in a semi-arid area in northeast Brazil. Five individuals of each species were analyzed.

Species	Average ± SE		
	C	N	C:N
Poincianella pyramidalis	214.72 ± 14.28	13.24 ± 0.34	29.17 ± 1.72
Mimosa spp.	218.31 ± 8.40	12.53 ± 0.40	17.95 ± 0.97
Croton spp.	218.15 ± 9.00	9.55 ± 0.54	17.03 ± 0.87
